# Effects of ursodeoxycholic acid on the gut microbiome and colorectal adenoma development

**DOI:** 10.1002/cam4.1965

**Published:** 2019-01-16

**Authors:** Talima Pearson, J. Gregory Caporaso, Monica Yellowhair, Nicholas A. Bokulich, Megha Padi, Denise J. Roe, Betsy C. Wertheim, Mark Linhart, Jessica A. Martinez, Cherae Bilagody, Heidie Hornstra, David S. Alberts, Peter Lance, Patricia A. Thompson

**Affiliations:** ^1^ Pathogen and Microbiome Institute Northern Arizona University Flagstaff Arizona; ^2^ Department of Biological Sciences Northern Arizona University Flagstaff Arizona; ^3^ University of Arizona Cancer Center University of Arizona Tucson Arizona; ^4^ Department of Molecular and Cellular Biology University of Arizona Tucson Arizona; ^5^ Department of Nutritional Sciences University of Arizona Tucson Arizona; ^6^ Stony Brook School of Medicine Stony Brook New York

**Keywords:** bile acid, colorectal adenoma, colorectal cancer, gut microbiome, Ursodeoxycholic acid

## Abstract

It has been previously reported that ursodeoxycholic acid (UDCA), a therapeutic bile acid, reduced risk for advanced colorectal adenoma in men but not women. Interactions between the gut microbiome and fecal bile acid composition as a factor in colorectal cancer neoplasia have been postulated but evidence is limited to small cohorts and animal studies. Using banked stool samples collected as part of a phase III randomized clinical trial of UDCA for the prevention of colorectal adenomatous polyps, we compared change in the microbiome composition after a 3‐year intervention in a subset of participants randomized to oral UDCA at 8‐10 mg/kg of body weight per day (n = 198) or placebo (n = 203). Study participants randomized to UDCA experienced compositional changes in their microbiome that were statistically more similar to other individuals in the UDCA arm than to those in the placebo arm. This reflected a UDCA‐associated shift in microbial community composition (*P* < 0.001), independent of sex, with no evidence of a UDCA effect on microbial richness (*P* > 0.05). These UDCA‐associated shifts in microbial community distance metrics from baseline to end‐of‐study were not associated with risk of any or advanced adenoma (all *P* > 0.05) in men or women. Separate analyses of microbial networks revealed an overrepresentation of *Faecalibacterium prausnitzii* in the post‐UDCA arm and an inverse relationship between *F prausnitzii* and *Ruminococcus gnavus. *In men who received UDCA, the overrepresentation of *F prausnitzii* and underrepresentation of *R gnavus* were more prominent in those with no adenoma recurrence at follow‐up compared to men with recurrence. This relationship was not observed in women. Daily UDCA use modestly influences the relative abundance of microbial species in stool and affects the microbial network composition with suggestive evidence for sex‐specific effects of UDCA on stool microbial community composition as a modifier of colorectal adenoma risk.

## 1 INTRODUCTION

Western diet and lifestyle account for up to 80% of colorectal cancer (CRC) incidence.^1^ Several factors have been proposed to explain these associations including an influence of diet and lifestyle on the gut microbiome and effects of colonic bacteria in tumor development.^2^ The interplay between gut bacterial composition and host epithelium is established as an important determinant of local immune function, metabolism, and host health. This includes strong experimental evidence for an important role of the gut microbiome in susceptibility to gastrointestinal cancers.^3^, ^4^ In human studies, reported differences in the gut microbiome between healthy and colorectal tumor tissues, including microbial community composition, support disturbances in intestinal bacteria in association with CRC.^5^ This includes findings of dense colonies of bacteria (ie, biofilms) invading the mucus layer in association with colorectal adenomas and cancers, particularly in patients with right‐sided neoplasia, which in vitro exhibit tumor‐promoting effects.^6^ However, establishing a causal relationship between gut bacteria and colorectal neoplasia in humans has been elusive.

Strong evidence for an etiologic role for gut bacteria in colorectal tumorigenesis has been obtained from mouse model studies.^2^ For example, in the dextran sodium sulfate (DSS), inflammation‐accelerated azoxymethane (AOM) mouse model of CRC, antibiotic treatment prior to and during AOM injection and throughout DSS treatment was shown to reduce tumor size and number.^7^ Further, stool and bedding from tumor‐bearing mice transferred to germ‐free mice treated with AOM/DSS was shown to increase tumor size and number, with AOM/DSS treatment exhibiting effects directly on microbial community composition. Such findings support microbiome remodeling as an important component of an inflammation/carcinogen model of intestinal tumor development and progression.

Multiple hypotheses have been proposed to explain a role for gut bacteria in CRC.^8^ These include pro‐tumorigenic effects of secondary bile acids, such as deoxycholic acid (DCA) and lithocholic acid (LCA). Secondary bile acids are derived by anaerobic colonic bacterial bile salt hydrolase (BSH) and 7α‐dehydroxylation from excess primary bile acids that reach the colon after evading ileal reabsorption from the small intestine into the enterohepatic circulation.^2^, ^9^, ^10^, ^11^ The bile acid‐CRC association led to extensive investigation of ursodeoxycholic acid (UDCA), a synthetic bile acid with favorable effects on bile acid pools, including DCA‐lowering action,^12^ activity in mouse models to prevent colon cancer,^13^ evidence of lower CRC risk in patients receiving UDCA for other indications^14^, ^15^ and, more recently, evidence that dysbiosis in the gut microbiome of patients with primary biliary cirrhosis (PBC) may be modified by treatment with UDCA.^16^

Despite the preclinical and clinical promise of UDCA, in a large phase III placebo‐controlled, randomized trial of UDCA for the prevention of colorectal adenomatous polyps, no overall effect of UDCA on adenoma risk was demonstrated. However, there was evidence for reduced risk for development of adenomas with high‐grade dysplasia.^17^ Subsequently, it was shown that UDCA significantly reduced risk for large and advanced adenoma in men with a positive trend toward larger and more advanced adenoma in women; findings that implicate sex as a modifier of UDCA activity in the colon.^18^ More recently, evidence for sexual differences in bile acid metabolism in mice^19^ and bile acid effects on gut bacterial composition^20^ have emerged. This prompted us to consider UDCA effects on the microbiome with specific attention to differences between men and women that might explain the gender disparity observed in secondary analyses of the adenoma prevention trial. Here, we present findings of UDCA effects on the fecal microbiome and results of exploratory analyses relating microbiome changes with UDCA to adenoma outcomes using data derived from archival paired stool specimens.

## 2 MATERIALS AND METHODS

### 2.1 Study group, sample collection, study design

Microbiome analyses were conducted on the fecal samples of a subset of subjects who consented to stool collection during their participation in the Phase III Chemoprevention Trial of Ursodeoxycholic Acid (UDCA) for the prevention of colorectal adenomatous polyps. Details of the trial are reported elsewhere.^21^, ^22^ Briefly, eligible individuals had at least one colorectal adenoma with a diameter of ≥3 mm removed during a colonoscopy performed no longer than six months prior to registration. In total, 1285 individuals were randomized to UDCA in a daily dose of 8‐10 mg/kg of body weight (n = 661) or matching placebo (n = 624); 1192 (613 UDCA and 579 placebo) participants completed the trial. Use of low‐dose aspirin was permitted up to 81 mg daily, for which randomization was stratified. The primary trial endpoint was colorectal adenoma, defined as the occurrence of one or more adenomas at colonoscopy performed at least 6 months after the initial qualifying colonoscopy. Advanced adenomas were defined as those with high‐grade dysplasia, villous/tubulovillous histology, or a diameter ≥1 cm.^18^ All stools passed over a 72‐hour period were collected in a single metal container on ice. Pooled 72‐hour samples were transported at 4°C to the laboratory where fecal solid was separated from fecal water as previously described.^21^, ^22^ Separated fecal water and solid stool were stored at −80°C for an average of 15 years until processing for microbial DNA.

For the current study, only participants with paired baseline (preintervention with UDCA or placebo) and end‐of‐study microbiome sequence data and adenoma outcome data were included. A total of 401 participants (198 UDCA and 203 placebo) with paired samples generated 802 samples for analysis.

### 2.2 DNA extraction

DNA was extracted from thawed stool samples using the QIAamp DNA Stool Mini Kit protocol (Qiagen Inc, Valencia, CA) according to the manufacturer's instructions without modifications. Briefly, 200 mg of feces was placed in a sterile, round‐bottom 2 mL tube containing 1.4 mL ASL lysis buffer. The homogenate was pelleted and incubated with InhibitEX to adsorb inhibitors. Proteinase K and Buffer AL were added to the supernatant to digest proteins. The DNA was bound to a spin column filter, and impurities were washed from the sample using 96%‐100% ethanol and proprietary Buffer AW2. All samples were eluted in 200 μL AE buffer and stored at −80°C until use in PCR.

### 2.3 PCR and sequencing

PCR of the V4 region of the 16S rRNA gene and sequencing were performed on the Illumina MiSeq platform following the original Earth Microbiome Project protocols (http://www.earthmicrobiome.org/protocols-and-standards/) originally described by Caporaso et al^23^ Sequencing was performed using paired‐end 150 base reads. Data presented are based on forward reads only, as 150 base paired‐end reads are too short to assemble for many organisms, and therefore can result in systematic bias against detecting taxa with shorter 16S rRNA gene sequences (since reads that cannot be assembled are not included in current paired‐end bioinformatics workflows). The choice of paired‐end vs single‐end analysis has been shown to result in little practical difference for human microbiome studies.^24^

### 2.4 Bioinformatics

Microbiome bioinformatics were performed with QIIME^25^ 2 2017.4, a plugin‐based system that, in some cases, wraps other microbiome analysis methods. Briefly, raw sequence data were demultiplexed and quality filtered using the q2‐demux plugin followed by denoising with DADA2^26^ (via q2‐dada2) to identify all observed amplicon sequence variants (ASVs)^27^ (ie, 100% operational taxonomic units [OTUs]). All ASVs were aligned with mafft^28^ (via q2‐alignment) and used to construct a phylogeny with fasttree2^29^ (via q2‐phylogeny). Alpha‐diversity metrics (observed OTUs and Faith's Phylogenetic Diversity^30^—measures of microbiome richness), beta diversity metrics (weighted UniFrac,^31^ unweighted UniFrac,^32^ Jaccard distance, and Bray‐Curtis dissimilarity—measures of microbiome composition dissimilarity), and Principle Coordinate Analysis (PCoA) were estimated using q2‐diversity after samples were rarefied (ie, subsampled without replacement) to 900 sequences per sample. A total of 900 sequences per sample were chosen as our rarefaction depth to retain all paired samples, as samples with fewer sequences than the rarefaction depth are excluded from downstream diversity analyses (Table S2 for details on the number of sequences obtained per sample). To ensure that this rarefaction depth was not too low, for each of our four beta diversity metrics, we tested distance matrices derived from feature tables rarefied at 900 sequences per sample and 10 000 sequences per sample for correlation using Mantel tests. We observed that these were significantly correlated for all metrics, suggesting that downstream conclusions should be robust to this rarefaction depth (Jaccard: *ρ* = 0.51, *P* < 0.001; Bray‐Curtis: *ρ* = 0.99, *P* < 0.001; unweighted UniFrac: *ρ*: 0.73, *P* < 0.001; weighted Unifrac: *ρ*: 0.99, *P* < 0.001; n = 675 pairwise comparisons for each test). We therefore opted to retain more samples (and therefore subjects) in our analysis, rather than more sequences. Taxonomy was assigned to ASVs using the q2‐feature‐classifier^33^ classify‐sklearn naïve Bayes taxonomy classifier against the Greengenes 13_8 99% OTUs reference sequences.^34^ This classifier was recently shown to achieve similar precision and recall to the RDP classifier^35^ at the genus level on 15 mock community data sets.

### 2.5 Statistics

The subjects included in this analysis were those who self‐selected to provide stool samples pre‐ and posttreatment. Differences in baseline characteristics between the subset of participants whose stools were analyzed in the current study and the parent trial, or between treatment arms, were tested using chi‐square tests for categorical variables and *t* tests or Wilcoxon rank‐sum tests for continuous variables. The difference between the freezer storage time in each treatment arm was tested using a linear mixed effects model, to account for the correlation induced by the baseline and end‐of‐study samples from the same subject. The association between freezer storage time and microbiome composition was tested using a Spearman correlation coefficient for baseline and end‐of‐study samples separately.

To test for differences in microbiome composition, we performed PCoA based on four distance metrics (weighted UniFrac, unweighted UniFrac, Bray‐Curtis, and Jaccard). Beta diversity metrics differ widely in the types of differences they detect, so we chose these four metrics as representatives of different ways of comparing community composition: Jaccard only reports differences in the presence or absence of ASVs; Bray‐Cutis reports differences in the presence, absence, and abundance of ASVs; unweighted UniFrac reports differences in the presence or absences of ASVs while up‐weighting differences in ASVs that are distantly evolutionarily related; and weighted UniFrac reports differences in the presence, absence, and abundance of ASVs while up‐weighting differences in ASVs that are distantly evolutionarily related.

Components of variance were used to estimate the between‐patient vs within‐patient intraclass correlation coefficient for each microbiome measure. We then computed the change (in direction and magnitude) in the first principal coordinate axis (PC1) for each subject between their pretreatment and posttreatment samples using q2‐longitudinal.^36^ The average change in PC1 for each treatment group, overall and stratified by sex, was tested for difference from zero using a one‐sample *t* test with Benjamini‐Hochberg false discovery rate (FDR) correction.^37^ We additionally applied pairwise tests to determine if UDCA treatment was associated with changes in gut microbial community richness (ie, changes in the number of bacterial taxa present in the community). This was performed by comparing change in Observed OTUs and Faith's Phylogenetic Diversity on a per‐subject basis in the two treatment groups.

To create microbial networks, we started by filtering out infrequent ASVs (those observed in fewer than 33% of the samples) and then computed correlations using an ensemble method composed of Spearman correlation, Pearson correlation, and SparCC. Edges that had *P* ≤ 0.001 with all three methods, and with correlation coefficients of the same sign with all three methods, were classified as highly significant.

Networks were created by representing the ASVs as nodes and using the SparCC correlation coefficient to define edge weights. To compare the postplacebo and post‐UDCA network, we used two methods. We first ran ALPACA (ALtered Partitions Across Community Architectures), a method for globally comparing a baseline and perturbed network to find modules (clusters) of increased edge density.^38^ ALPACA was originally formulated for bipartite graphs with positive edge weights. In order to apply it to microbial networks, we ran ALPACA using the unipartite version of the differential modularity score and defined the edge weights to be the absolute value of the SparCC coefficients. Nodes were ranked by their contribution to the differential modularity.^38^ This analysis gives the same level of importance to both positive and negative correlations, thus incorporating both types of microbial interactions equitably, but cannot functionally distinguish between them.

We also created a “differential network” by directly subtracting the postplacebo correlations from the post‐UDCA correlations and keeping only the positive subtracted edge weights. The Louvain method was then used to perform community detection on the differential network, and nodes were ranked by their contribution to the modularity.^39^ This analysis retains information about the sign of the interaction and clusters nodes into groups of OTUs that have increased positive interaction after UDCA.

We performed ANCOM^40^ and Wilcoxon signed‐rank tests comparing species abundance at baseline and end‐of‐study in both UDCA‐treated and placebo groups. ANCOM tests were performed to assess differences within the whole bacterial community in each arm separately. Wilcoxon signed‐rank tests were additionally performed on 18 individual bacterial genera, the order *Bifidobacteriales*, and the ratio of the *Firmicutes *to *Bacteroidetes *phyla abundances, all of which have been previously associated with CRC or its risk factors.

Associations between change in each microbiome measure and adenoma outcome (any adenoma or advanced adenoma) were tested in each arm separately using Poisson regression, adjusted for sex, age, aspirin use, baseline microbiome measure, and an indicator for whether a participant's paired baseline, and end‐of‐study DNA samples were processed in different batches. Potential interactions between microbiome measures and UDCA on recurrence were tested using likelihood ratio tests. These statistical tests were performed with Stata 14.2 (StataCorp, College Station, TX).

## 3 RESULTS

### 3.1 Participant characteristics

Characteristics of the 401‐participant subgroup with complete sequence data and adenoma outcome status were compared to participants in the parent trial not included in the microbiome study, by treatment assignment (Table 1). Placebo arm participants in the microbiome study subgroup had fewer aspirin users (chi‐square test, *P = *0.004), greater mean diameter of their largest adenomas (Wilcoxon rank‐sum test, *P = *0.040), and greater numbers of adenomas at baseline (Wilcoxon rank‐sum test, *P = *0.004) than placebo participants who were not included in the microbiome study. Microbiome study participants receiving UDCA included more men than parent‐trial participants not included in the microbiome study (chi‐square test, *P* = 0.016). Among participants in the microbiome study, the UDCA arm included more males (chi‐square test, *P* = 0.024) and more aspirin users (chi‐square test, *P* = 0.003) than the placebo arm.

**Table 1:** Baseline characteristics of participants in the subsample compared to the parent trial, by treatment arm

Variable	Placebo arm		UDCA arm	
	Subsample (*n* = 203)	Parent trial (*n* = 421)	Subsample (*n* = 198)	Parent trial (*n* = 463)
Age, mean ± SD	66.5 ± 8.0	66.3 ± 8.5	66.2 ± 8.9	66.0 ± 8.6
Male, *n* (%)	133 (65.5)	280 (66.5)	150 (75.8)	307 (66.3)
White, *n* (%)	188 (94.0)	388 (93.7)	189 (96.9)	426 (94.3)
Education (years), mean ± SD	13.9 ± 2.3	14.1 ± 2.3	14.1 ± 2.3	13.9 ± 2.2
Ever smoker, *n* (%)	134 (69.1)	293 (71.6)	125 (64.1)	314 (69.8)
Current smoker, *n* (%)	21 (10.3)	57 (13.5)	23 (11.6)	56 (12.1)
BMI (kg/m^2^), mean ± SD	28.4 ± 4.7	28.1 ± 4.8	28.0 ± 4.9	28.1 ± 4.8
Aspirin use, *n* (%)	39 (19.2)	127 (30.2)	64 (32.3)	124 (26.8)
Family history of CRC, *n* (%)	66 (32.5)	115 (27.3)	57 (28.8)	111 (24.0)
Previous polyp, *n* (%)	77 (40.1)	189 (48.6)	94 (48.7)	209 (48.1)
Largest adenoma (mm), mean ± SD; median	9.6 ± 6.3; 8	8.4 ± 5.4; 7.5	8.9 ± 5.4; 8	8.7 ± 5.4; 8
Number of adenomas, mean ± SD; median	1.6 ± 0.9; 1	1.5 ± 0.8; 1	1.7 ± 1.1; 1	1.6 ± 0.9; 1
Proximal adenomas, *n* (%)	113 (55.7)	227 (54.2)	112 (56.6)	260 (56.3)
Villous component to adenoma, *n* (%)	46 (22.7)	78 (18.5)	33 (16.7)	106 (23.0)
High‐grade dysplasia, *n* (%)	21 (10.3)	35 (8.3)	19 (9.6)	38 (8.2)

Missing data: race, *n* = 24 (1.9%); education, *n* = 29 (2.3%); ever smoker, *n* = 37 (2.9%); BMI, *n* = 29 (2.3%); previous polyp, *n* = 76 (5.9%); largest adenoma, *n* = 1 (0.1%); proximal adenoma, *n* = 3 (0.2%); villous histology, *n* = 2 (0.2%).

### 3.2 Microbiome composition is not correlated with storage time

After separation from fecal water, solid stool samples were stored at −80°C for varying lengths of time before microbiome sequencing. Baseline samples were stored for an average of 17.2 ± 1.1 years, and end‐of‐study samples were stored for an average of 14.6 ± 1.1 years. There was no significant difference in storage time by treatment arm (*P* = 0.22). Furthermore, no significant correlations were observed between storage time and any of the diversity metrics at baseline or end‐of‐study. Lack of evidence for storage‐time effects on these measures is in agreement with published studies supporting long‐term freezing as an effective preservation method for studies of microbiome composition.^41^

### 3.3 Microbiome changes in response to UDCA treatment

**Figure 1:**
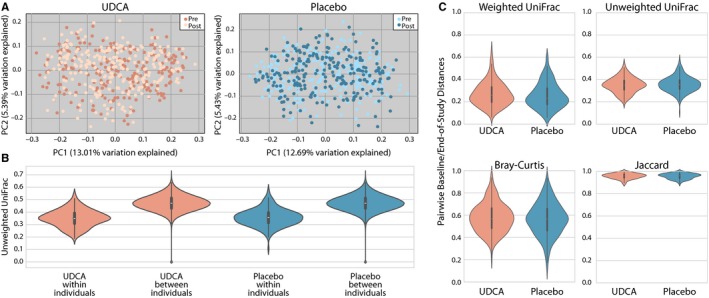
A, PCoA plots for UDCA and placebo groups with pre‐ and postsamples (light and dark, respectively). B, Violin plots illustrate the full distribution of data for different values of unweighted UniFrac distances within and between individuals. Markers for the median (center point), interquartile range (box), and 1.5 interquartile range (whiskers) are included. Distances within individuals are significantly less than distances between individuals. C, Violin plots depict the magnitude of change in microbiome composition between baseline and end‐of‐study in UDCA and placebo groups. The magnitude of change did not differ significantly between the treatment groups for any of these metrics

Principle coordinate analysis based on unweighted UniFrac distance between samples showed no clear differences between baseline and end‐of‐study microbial communities in either treatment group (Figure 1A). Distances between paired samples from the same subject were smaller than distances between samples from different subjects in both treatment groups (Figure 1B). Intraclass correlation coefficients estimated separately for each of the four beta diversity metrics ranged from 0.50 to 0.68 for the placebo group, and from 0.39 to 0.73 for the UDCA group (Figure 1B). There was no clear pattern of change between the UDCA and placebo arms by these four broad measures of microbial community composition (*U* = 19 292.00, *P* = 0.244; Figure 1C), suggesting that both treatment groups experience changes in their microbiomes between baseline and end‐of‐study.

**Figure 2:**
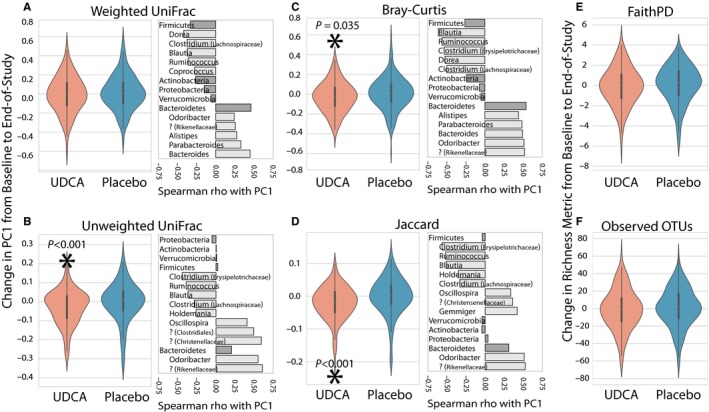
Pairwise changes in PC1 between baseline and end‐of‐study samples (left panels) and correlation with taxonomic changes (right panels) shown for phyla (dark gray bars) and genera (light gray bars). Question marks indicate unknown genera and include the most specific known taxonomic association in parentheses. A‐D, Change in PC1 for microbial community distance metrics in each treatment arm. E and F, Change in microbial community richness metrics in each treatment arm. Statistically significant comparisons are indicated with an asterisk and *P*‐value

Given the apparent similarity in microbiome composition between UDCA and placebo groups according to beta diversity metrics, we next tested whether within‐group similarities in microbiome changes could distinguish the UDCA and placebo groups. Paired one‐sample t tests were used to identify consistent changes across individuals in four microbial community distance metrics (Figure 2A‐D) and two microbial community richness metrics (Figure 2E,F). In this analysis, UDCA treatment was associated with a shift in microbial community distance metrics according to PC1 of unweighted (ie, qualitative) UniFrac (*t* = −4.393, *P* < 0.001) distance, and PC1 of both qualitative (Jaccard: *t* = −5.697, *P* < 0.001) and quantitative (Bray‐Curtis; *t* = −2.699, *P* = 0.035) nonphylogenetic metrics. By definition, PC1 explained the largest percent variation in the data for each of these metrics. Percent variation for the first three axes of each PCoA computed on all 802 samples are as follows: weighted UniFrac PC1: 33.6, PC2: 9.8, PC3: 5.7; unweighted UniFrac PC1: 12.2, PC2: 4.8, PC3: 4.4; Bray‐Curtis PC1: 6.8, PC2: 4.6, PC3: 4.0; Jaccard PC1: 1.0, PC2: 0.5, PC3: 0.5. We focused our analyses on PC1 only since it explains the most variation in our data, and to avoid multiple comparisons errors. These shifts were not observed in the placebo arm. These results suggest that while gut microbial community composition changed by a similar degree in the UDCA and placebo groups according to beta diversity metrics (Figure 1C), individuals in the UDCA arm experienced changes that were more similar to each other (ie, were UDCA‐associated) than those in the placebo arm (Figure 2B‐D). For gut microbial community richness (ie, changes in the number of bacterial taxa present in the community), Observed OTUs and Faith's Phylogenetic Diversity were computed on a per‐subject basis in each arm (Figure 2E,F). The average change was not significantly different from zero in either arm for either measure (all *P* > 0.05). Therefore, despite UDCA‐associated changes in overall community composition, we found no evidence that UDCA treatment significantly altered gut microbial community richness.

**Figure 3:**
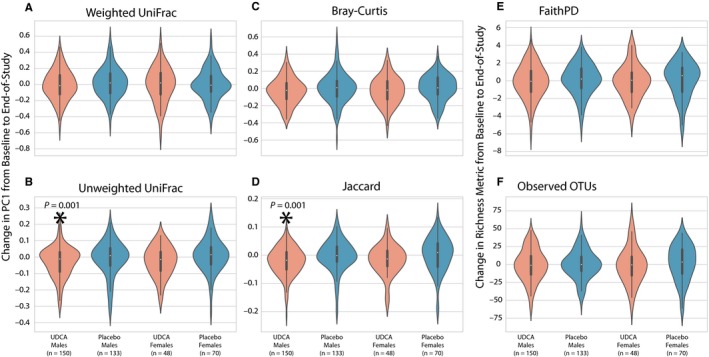
Pairwise changes between baseline and end‐of‐study samples stratified by treatment arm and sex. A‐D, Change in PC1 for microbial community distance metrics. E and F, Change in microbial community richness metrics. Statistically significant comparisons between treatment arms are indicated with an asterisk and *P*‐values

Because UDCA treatment was shown to be protective against the development of advanced adenomas in males but not females in the parent trial,^18^ we next explored results stratified by sex (Figure 3A‐F). Using a pairwise approach, two of the six microbiome measures showed a statistically significant change with UDCA treatment in males (unweighted UniFrac [*t* = −4.393, *P* < 0.001] and Jaccard [*t* = −5.234, *P* < 0.001]). For females, none of the metrics showed a significant change with UDCA. This could be interpreted as being consistent with the findings in the parent trial, but it is important to note that the mean change in PC1 was the same in the male and female groups, so the lack of statistical significance in females may be due to the smaller sample size (48 women vs 150 men). No sex‐specific changes were observed in the placebo arm.

With the observed changes in community composition in response to UDCA treatment, we were interested in identifying bacterial taxa that exhibited abundance changes. ANCOM tests indicated that no bacterial genera or ASVs consistently differed between baseline and end‐of‐study measurements in the placebo group, or when comparing posttreatment samples across the placebo and UDCA groups. In the UDCA treatment arm, ANCOM tests on all ASVs showed that the relative abundance of Faecalibacterium decreased between baseline and end‐of‐study. Paired Wilcoxon signed‐rank tests were additionally performed on 18 individual bacterial taxa that contain species or strains previously associated with CRC^42^ (Table S1). Of these, Streptococcus (FDR‐corrected *P* = 0.003), Escherichia (FDR‐corrected *P* = 0.003), and Bilophila (FDR‐corrected *P* = 0.012) were found to have increased significantly, while Fusobacterium (FDR‐corrected *P* = 0.049) decreased in relative abundance between baseline and end‐of‐study in UDCA‐treated subjects. There were no significant changes for these genera in the placebo arm (all FDR‐corrected *P* > 0.05). We additionally tested whether the ratio of the Firmicutes to Bacteroidetes phylum abundances changed with treatment using Wilcoxon signed‐rank tests, but did not find evidence for this in either treatment group (UDCA: *W* = 10 369.5, FDR‐corrected *P* = 0.57; placebo: *W* = 9081.0, FDR‐corrected *P* = 0.13).

### 3.4 Microbiome network change in response to UDCA

**Figure 4:**
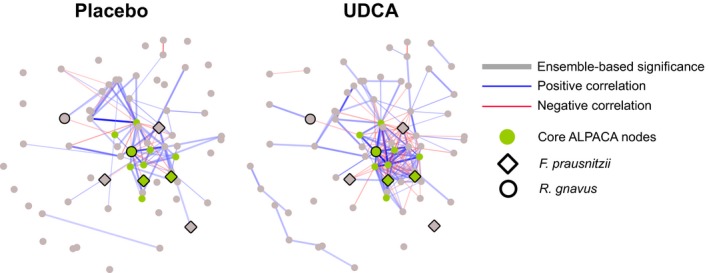
Microbial networks after placebo or UDCA treatment. Red edges indicate negative correlation and blue edges indicate positive correlation. Edge opacity is proportional to absolute value of SparCC correlation coefficient. Thick edges denote statistical significance of *P* < 0.001 by all three methods (Spearman, Pearson and SparCC; ie, edges that are significant by our ensemble method). Green nodes represent top ten ASVs from ALPACA (with stronger interactions in the UDCA group), and larger nodes with black border are annotated to *Faecalibacterium prausnitzii* and *Ruminococcus* *gnavus*.

While PCoA is powerful for reducing complex microbiome data to a few structure‐based dimensions, intra‐microbiome interactions and their associations with explanatory variables can be difficult to uncover as statistical testing is generally conducted using one microbiome feature (eg, ASV) at a time. As a result, discovery of more complex dynamics between the microbiome and factors like UDCA can be obscured. To address this, network analysis (or graph theory) offers a methodological framework to represent pairwise associations among microorganisms and provide insight into interactions among species that may be distinct from those involving their constituent members. We used the SparCC package to compute the correlation coefficient between every pair of ASVs that was observed in at least 33% (n = 264) of the samples. We employed this threshold to reduce data sparsity, a known issue with network analysis of microbiome data.^43^ We next used the resulting correlation coefficients as edge weights to construct microbial networks across all subjects before and after intervention. We then applied ALPACA,^38^ a method for comparing two networks to find clusters of nodes which interact more in one context than another. A cluster represents a set of nodes that have stronger connections between themselves than with other nodes in the network. Comparing the postplacebo and post‐UDCA networks using ALPACA, we found one group of ASVs that are more strongly interacting (ie, exhibit a higher absolute value of correlation) after UDCA treatment than after placebo treatment (Figure 4). The top ten of these ASVs are annotated to several different genera, including Ruminococcus, Blautia, and Faecalibacterium (Table S3). We observed an overrepresentation of *Faecalibacterium prausnitzii* among the top‐ranked ASVs in the module (*P* = 0.011, Wilcoxon rank‐sum test).

**Figure 5:**
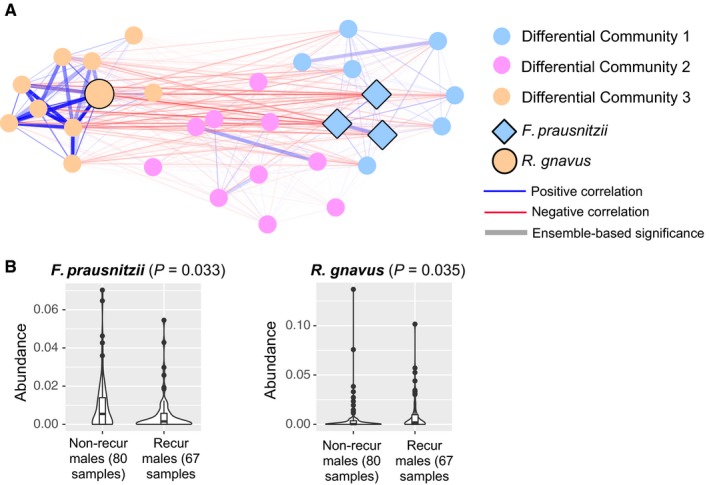
A, Differential network communities. The edges represent significant SparCC correlations after UDCA treatment, with red indicating negative correlation and blue indicating positive correlation. Opacity of edges is proportional to the absolute value of SparCC correlation coefficients. Thicker edges denote statistical significance of *P* < 0.001 by all three methods (Spearman, Pearson and SparCC; ie, edges that are significant by our ensemble method). Nodes represent the top thirty ASVs in the differential network communities, with the node color indicating community membership. Nodes with black borders are annotated to *Faecalibacterium* *prausnitzii* or *Ruminococcus gnavus*. B, Violin plots showing the abundance distribution of two ASVs annotated to *F prausnitzii* and *R gnavus* in males with or without recurrence after UDCA treatment. *P*‐values computed using Wilcoxon rank‐sum test.

It is evident that both the placebo and UDCA networks contain a mix of positive and negative correlations, showing that different microbes play different roles in the ecosystem (Figure 4). To examine the effect of UDCA on a more granular level, we created a “differential network” where each edge represents the increase in the absolute value of the SparCC correlation coefficient (suggesting stronger interactions) upon UDCA treatment. The second biggest hub in the differential network is a member of *F prausnitzii*, reinforcing a potentially important interactive role for this species, with a positive correlation with the other *F prausnitzii *ASVs and negative correlations with *Ruminococcus gnavus *and many other members. We then used the Louvain method to partition the differential network into three communities (Figure 5A).^39^ Differential Community 1 (DC1) contains three ASVs of *F prausnitzii *that are positively correlated with other members of DC1. These *F prausnitzii *ASVs (and other members of DC1) are negatively correlated with DC3 which includes Blautia and *R gnavus.*

### 3.5 Change in microbiome and adenoma risk

We next assessed whether UDCA‐associated changes in community composition or network structure, when controlled for the baseline value, were associated with adenoma development. We found no evidence that change in any of the four microbial community distance metrics from baseline to end‐of‐study was associated with adenoma risk in either treatment arm (all *P* > 0.05) even after considering effects by sex separately. For the specific ASVs that were found to increase or decrease with UDCA treatment (ie, Streptococcus, Escherichia, Bilophila, and Fusobacterium), we found no evidence of association between any of these ASVs and adenoma outcome in either the placebo or UDCA arm (all *P* > 0.05).

Microbial network analysis revealed no significant associations between the top correlated microbial species post‐UDCA and adenoma risk (data not shown). However, in exploratory analyses restricted to men who received UDCA treatment, two ASVs that were annotated to the species level (*R gnavus *and *F prausnitzii*) appeared to be differentially abundant between men with and without adenoma development (Figure 5B). These associations were not observed in women (data not shown).

This prompted us to test the association between change in *R gnavus* or *F prausnitzii* and adenoma development across the intervention groups, controlling for potential confounders. After adjusting for age, aspirin use, and the baseline level of the species, a positive change (increase) in *R gnavus* was significantly associated with higher adenoma risk in men in the UDCA arm (log binomial regression, *P* = 0.009). Conversely, in a similar model for men in the UDCA arm, a positive change (increase) in *F prausnitzii* was significantly associated with lower adenoma risk (log binomial regression, *P* = 0.034). Men in the placebo arm had a significantly greater increase in *R gnavus* between the two time points than men in the UDCA arm (Wilcoxon rank‐sum test, *P* = 0.015), but no significant difference in the degree or direction of change in *F prausnitzii* (Wilcoxon rank‐sum test, *P* = 0.130). Within the UDCA arm, and similar to the community composition, we did not observe any differences in *R gnavus* or *F prausnitzii *change in men when compared to women (both *P* > 0.2), though small sample sizes in each of these analyses limit our ability to detect such differences. Overall, there were no significant associations between *R ganvus* or *F prausnitzii* in the placebo arm and adenoma outcomes for men or for women.

## 4 DISCUSSION

Our initial analysis of community composition revealed changes over time for both the UDCA and placebo treatment groups, but no overall posttreatment community similarities (Figure 1). However, paired analysis of microbial composition changes illustrated significant similarities in the community shifts in the UDCA but not the placebo group (Figures 2and 3). This suggests that nonrandom changes occurred in the microbiomes of individuals in the UDCA group, but not placebo. Changes in microbial richness were not seen in either group, implying that compositional microbiome changes associated with UDCA treatment reflected alterations in the relative abundance and presence of microbial taxa, but not significant changes in the total number of taxa that were present.

Bacteroidetes and Firmicutes are two dominant microbial phyla comprising the gut microbiome. These common bacteria in the human gut and their ratio to each other have been suggested to reflect dietary pattern and overall balance of the gut microbiome. For example, a high Firmicutes to Bacteroidetes ratio has been associated with consumption of the Western diet^17^ and with adverse metabolic changes that occur with obesity.^44^, ^45^ In contrast, a low Firmicutes to Bacteroidetes ratio has been associated with reduced gut biodiversity^46^ and observed in patients with inflammatory bowel disease.^47^ We observed Bacteroidetes and Firmicutes abundances to be positively and negatively correlated with PC1, respectively, in our PCoAs. On average, by all four metrics, we observe a decrease in PC1 value with UDCA treatment. Although this decrease is significant for three of the four metrics, in all cases it represents a small shift in community composition. In these PCoAs, a decrease in PC1 is correlated with an increase in Firmicutes and a decrease in Bacteroidetes abundance, but as the observed changes in PC1 are small, any average change in the abundance of these specific two phyla is likely negligible. This is supported by the change in the ratio of these taxa with treatment being insignificant in our Wilcoxon‐based differential abundance tests.

UDCA‐associated increases in species of Streptococcus, Escherichia, and Bilophila and decreases in Fusobacterium are notable in the context of reported associations between different members of these genera and CRC. An increase in Bilophila is biologically consistent with earlier studies showing that UDCA led to increases in the levels of DCA in aqueous and solid stool fractions, with evidence that UDCA may enhance fecal bile acid levels through inhibitory effects on 7‐α‐dehydroxylation of cholic acid. As such, expansion of Bilophila would be expected, but perhaps not desirable, given pro‐inflammatory effects of *Bilophila wadsworthia* in mice. Increases in members of the genera Streptococcus and Escherichia with UDCA may similarly reflect response to changes in the bile acid pool in stools of UDCA subjects.

Network analyses suggested that UDCA impacts microbial communities by increasing the co‐occurrence of certain taxa (Ruminococcus, Blautia, and Faecalibacterium and *F prausnitzii*). Co‐occurrence may be indicative of interaction, but may also be a result of UDCA selecting for or against certain taxa (so importantly does not imply a functional interaction between co‐occurring taxa). While we saw no significant overall associations between bacterial networks and adenoma risk, we did find evidence that the abundances of certain taxa that were identified as interesting in our network analyses were associated with adenoma risk in males. This includes decreased in *R gnavus *and increased *F prausnitzii* abundance. These results support a chemopreventive role of UDCA in males that may in part be due to inhibition of *R gnavus *reported to be enriched in patients with inflammatory bowel disease^48^ and increased prevalence of *F prausnitzii* where specific strains have been shown to suppress experimental colitis. The small sample size of women may have precluded us from observing a significant differential effect of UDCA on the gut microbiome by sex. The secondary finding from the original trial, that UDCA may reduce risk for adenoma in men but not women, therefore still needs further investigation, first for confirmation and second to determine whether it can be explained by a microbiome‐associated mechanism.

Longitudinal variation of the gut microbial community within individuals is expected,^49^ and the degree of variation differs between individuals.^50^, ^51^ This variation, along with the high intraclass correlation coefficients observed in our study and evidence that components of the microbiome are highly individualized,^52^ is a significant limiting factor for the detectability of subtle but potentially important effects of medical treatment on the microbiome. Nonetheless, treatment with the therapeutic bile acid UDCA, for three years led to demonstrable changes in the fecal microbiome that, in men, are plausibly related to UDCA action to inhibit adenoma development.

## CONFLICTS OF INTEREST

The authors declare no conflicts of interest.

## Data Availability

Raw sequence data are currently being deposited in the Qiita microbiome meta‐analysis repository, and the ENA sequence read archive. Accession numbers will be included in the final version of this manuscript, after they have been generated. Software and data are available under open source/open access licenses via https://github.com/caporaso-lab/crc-udca2.
