# Supplementary figures and images for: Effects of ursodeoxycholic acid on the gut microbiome and colorectal adenoma development
Source: Cancer Med. 2019 Jan 16;8(2):617–28. doi: 10.1002/cam4.1965 (PMC6382922; doi:10.1002/cam4.1965)

(a) weighted UniFrac PCoA

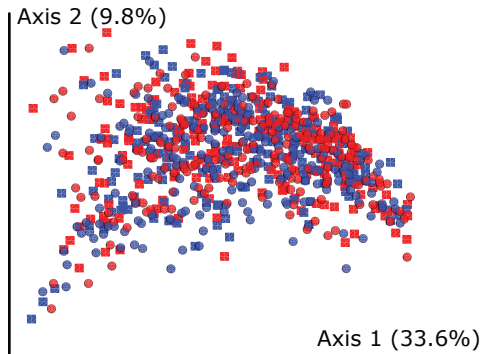

(b) unweighted UniFrac PCoA

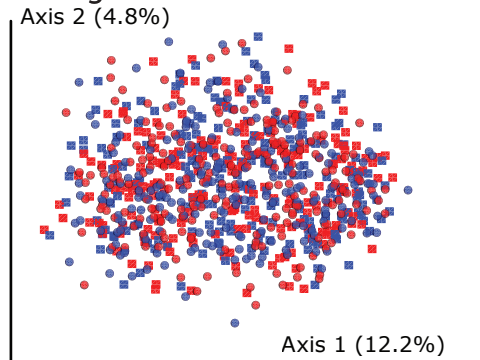

(c) Bray-Curtis PCoA

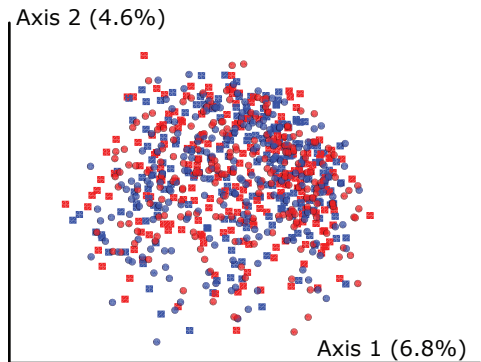

(d) Jaccard PCoA

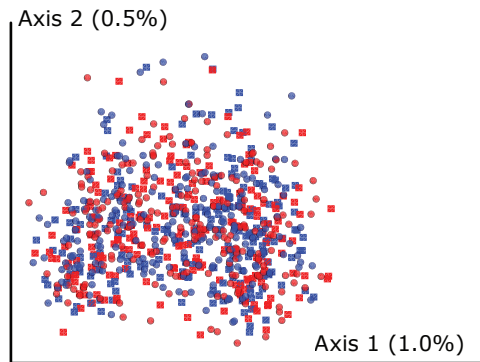

Supplement: Supplementary file 1 [file CAM4-8-617-s001.pdf]
